# Enhancement of phytolith-occluded carbon accumulation of Moso bamboo response to temperatures elevation and different fertilization

**DOI:** 10.3389/fpls.2023.1144961

**Published:** 2023-03-13

**Authors:** Lijun Liu, Scott X. Chang, Chengpeng Huang, Yuyou Zhi, Yang Jie, Xiuling Yu, Peikun Jiang

**Affiliations:** ^1^ State Key Laboratory of Subtropical Silviculture, Zhejiang A&F University, Lin’an, Zhejiang, China; ^2^ School of Environmental and Resources Science, Zhejiang A&F University, Lin’an, Zhejiang, China; ^3^ Department of Renewable Resources, University of Alberta, Edmonton, AB, Canada; ^4^ Department of Bamboo Research, Fujian Academy of Forestry, Fuzhou, Fujian, China

**Keywords:** phytolith, PhytOC, silicon fertilizer, soil microbe, temperature, land management

## Abstract

The accumulation of phytolith-occluded carbon (PhytOC) in Moso bamboo could be a novel long-term carbon sequestration strategy. The objective of this study was to investigate the effects of temperature change and different fertilization on PhytOC accumulation. The pot experiment was established with different fertilization (including control (CK), nitrogen fertilizers (N), silicon fertilizers (Si), and a combination of nitrogen and silicon (NSi)) under high- and low-temperature. Despite the different fertilization, the PhytOC accumulation of the high-temperature group increases by 45.3% on average compared with the low-temperature group, suggesting higher temperature is greatly beneficial to the PhytOC accumulation. Fertilization significantly increases the accumulation of PhytOC (increased by 80.7% and 48.4% on average for the low- and high-temperature group, respectively) compared with CK. However, the N treatment increased both Moso bamboo biomass and PhytOC accumulation. The difference in the accumulation of PhytOC in Si and NSi was insignificant, indicating the combination of N and Si didn’t bring extra benefit to PhytOC accumulation compared to Si fertilizer alone. These results indicated the application of nitrogen fertilizer is a practical and effective method for enhancing long-term carbon sequestration for Moso bamboo. Based on our study, we conclude that global warming poses a positive effect on promoting the long-term carbon sequestration of Moso bamboo.

## 1 Introduction

The continuous change of climate has appeared to accompany the development of the pre-industrial era (Redlin and Gries, 2021). Plants play an important role in carbon sinks by absorbing carbon dioxide (CO_2_) and mitigating climate change (Bonan, 2008; Li, 2022; Liu et al., 2022). However, some organic carbon that is temporarily fixed by plants may return to the atmosphere through biochemical cycles. Therefore, long-term carbon sequestration strategies for terrestrial ecosystems need to be further explored. As a typical silicon accumulator, bamboo produces a large amount of phytolith during the growth process. The phytoliths are amorphous silica particles that are deposited during plant growth (Ma and Yamaji, 2006). During the formation process, a fraction of organic carbon was occluded within the phytolith, which is called phytolith occluded carbon (PhytOC) (Parr and Sullivan, 2005). The PhytOC could be locked up in phytoliths for centuries or even longer periods without being returned to the atmosphere as quickly as other organic carbon components (Meunier, 1999; Blecker et al., 2006). Hence, the plant PhytOC sequestration mechanism could make a novel way in mitigating climate change (Song et al., 2016). The area of bamboo the forest is about 6.42 million hectares in China, among which the Moso bamboo (*Phyllostachys heterocycla* (Carr.) Mitford cv. *Pubescens* Mazel ex H.de leh.) forest covers the largest area, accounting for about 72.96% (MOF, 2019). Therefore, the Moso bamboo is of great potential for long-term carbon sequestration.

For years, many researchers have focused on enhancing PhytOC accumulation. Studies revealed that the accumulation of phytolith and PhytOC are related to biomass, which is achievable through fertilization (Song et al., 2013; Huang et al., 2020; Lu et al., 2021; Tan et al., 2021). Therefore, the promotion of PhytOC sequestration through fertilization has been frequently investigated by previous research (Song et al., 2013; Huang et al., 2015). Studies have demonstrated that nitrogen (N) fertilizer contributes to plants’ biomass and phytolith production (Zhao et al., 2016; Beard et al., 2018; Lu et al., 2021). In recent years, silicon fertilizer was widely applied to increase plants’ tolerance to biological and abiotic stresses (Li et al., 2006; Ma and Yamaji, 2006; Xie et al., 2014). As a typical silicon-accumulating plant, the application of silicon (the basic element of phytolith) is also beneficial for the accumulation of PhytOC in plant tissues (Guo et al., 2015; Sun et al., 2019; Huang et al., 2020). However, whether the combination of N and Si (NSi) fertilizer can enhance PhytOC production is still awaiting interpretation.

Under the background of global warming, the temperature continues to rise over the past years. Studies revealed that the elevation of temperature may enhance various physicochemical and biological processes, thereby promoting silicon uptake and accumulation within the plants (Hussain and Maqsood, 2011; Calatayud et al., 2016). Other studies indicated that the production of phytolith and PhytOC positively responds to silicon accumulation (Yang et al., 2015; Ying et al., 2016; Yang et al., 2018). However, whether the elevation of temperature directly promotes the accumulation of phytolith and PhytOC is still unclear.

Although the strategies for enhancing the accumulation of phytolith and PhytOC have been investigated extensively throughout the world. Few studies, however, have investigated the effects of fertilization on PhytOC accumulation under different temperatures. Moreover, the optimized management strategies for enhancing PhytOC sequestration when considering multiple factors are not available yet. The aims of this study were: 1) to investigate the effects of temperature elevation and different fertilization on the PhytOC sequestration in Moso bamboo leaves; 2) explore the possible mechanisms of PhytOC accumulation in Moso bamboo leaves and 3) propose a practical and cost-effective management method to enhance the long-term carbon sequestration for Moso bamboo forest.

## 2 Materials and methods

### 2.1 Experimental soils and bamboo seedlings

The bamboo soil was obtained from the Moso bamboo forest with 18 years of cultivation history in SuiYang forestry Centre, Lin ‘an, Zhejiang Province, China. The physicochemical properties of the potted soil are shown in **Table 1**. The Moso bamboo seedlings were one-year-old seedlings cultivated by Sichuan Emei Fuyu Seedling Corporation. The seedling’s provenance was from Guilin, Guangxi, China. The quality assurance of the Moso bamboo seedlings were strictly performed to make sure reliable results of this study. The detailed properties related to the quality of the Moso bamboo seedlings for different treatments is given in **Table 2**.

**Table 1:** Basic chemical properties of the pot soil.

pH	TN (g kg^-1^)	TC (g kg^-1^)	TK (g kg^-1^)	TP (g kg^-1^)	TSi (%)	AN (mg kg^-1^)
4.93	0.8	9.8	17.95	0.23	24.3	70.49

TN, total nitrogen; TC, total carbon; TK, total potassium; TP, total phosphorus; TSi, total silicon; AN, alkali-hydrolyzed nitrogen.

**Table 2:** Characters of Moso bamboo seedlings for different temperature groups and fertilization treatments.

Groups	Treatment	Height (cm)	DBH (cm)	Leave number
Low Temp	CK	34.86 (1.56) a	0.36 (0.03) a	7.75 (0.75) a
	N	35.64 (1.23) a	0.39 (0.04) a	7.91 (1.00) a
	Si	34.62 (1.19) a	0.35 (0.05) a	7.67 (1.07) a
	NSi	35.27 (1.36) a	0.38 (0.05) a	7.83 (1.02) a
High Temp	CK	35.62 (0.87) a	0.36 (0.06) a	7.55 (1.13) a
	N	35.29 (1.72) a	0.34 (0.05) a	7.50 (0.90) a
	Si	35.43 (1.36) a	0.33 (0.05) a	7.58 (0.90) a
	NSi	35.60 (1.39) a	0.34 (0.06) a	7.42 (0.67) a

This table shows the mean values of the height, diameter at breast height and leave number of Moso bamboo seedlings for different temperature groups and fertilization. DSB: Diameter at breast height. CK, N, Si, and NSi represent the level of fertilization at 0N 0Si kg/hm^2^, N157.5 kg/hm^2^, Si 315 kg/hm^2^, the combination of N157.5 kg/hm^2^ and Si 315 kg/hm^2^, respectively. The values in brackets represent the standard deviation. The letters following values indicate significant different between treatments (p< 0.05).

### 2.2 Pot experiment

The soil was placed in a pot for a weight of 10 kg/pot. Four fertilizer treatments were designed in the pot experiment. CK, no fertilizer applied); N, N fertilizer with CO (NH_2_)_2_ 1.52 g/pot (at the level of N 157.5 kg/hm^2^); Si, Si fertilizer with Na_2_SiO_3_·9H_2_O 6.62g/pot (at the level of Si 315 kg/hm^2^); NSi, N fertilizer with CO (NH_2_)_2_ 1.52 g/pot and Si fertilizer with Na_2_SiO_3_·9H_2_O 6.62g/pot (equivalent to N 157.5 kg/hm^2^ and Si 315 kg/hm^2^). For the pot of CK and N fertilizer, 2.47g Na_2_CO_3_ was added to equalize Na^+^ concentration. After the fertilizer is thoroughly mixed with the soil, three strong Moso bamboo seedlings with a height of about 30 cm were planted in each pot. Subtract 60% of leaf areas from each leaf to reduce transpiration and improve survival. Each treatment was repeated four times. Water every one or two days so that the soil water content is controlled to 80% of the field capacity. The experiment was conducted on the campuses of Fujian Academy of Forestry Sciences, Fuzhou (E:119.27; N:26.15) and Zhejiang A&F University, Lin’an (E:119.43; N:30.15) with an average temperature of 16.0 and 20.0 °C, respectively. The experimental period was from February 13^th^, 2021 to December 2^nd^, 2021.

### 2.3 Sample collection and determination

Bamboo leaves and soil samples were collected on July 10^th^, 2021, and December 2^nd^, 2021, for further analysis. The third leaf from the top of each branch in each basin and mixed evenly cleaned with pure water to remove surface stains and further cleaned with ultra-pure water, and then subjected to ultrasonic shock for 15min until the leaf surface was clean. Leaves were then defoliated at 105 °C for 20min, dried at 80 °C for 48 h to constant weight, and cut into pieces, pulverized evenly with a ball mill. At the same time, take soil samples of 0-20 cm with the soil harvester to avoid damaging the roots. Carefully take three times per pot and mix evenly. After air drying and impurity removal, the soil sample was passed through a 10-mesh sieve, and the fine roots were removed by electrostatic adsorption. Then a small amount of soil was ground through a 100-mesh sieve with an agate mortar. All seedlings’ leaves were harvested on December 2^nd^, 2021. Leaves were weighed separately after being cleaned and dried.

In the middle and end of the experiment, photosynthesis and transpiration were measured by the portable photosynthesis system tester (Licor6400, American). The timing was from 9 a.m. to 11 a.m., with clear weather conditions. Three branches with the same growth were selected for each potted seedling, and the third fully expanded leaf from the top of each branch was determined as the functional leaf, and the measurement was repeated 5 times. Phytoliths of Moso bamboo seedling leaves were extracted using microwave digestion with a modified Walkley-Black method and further examined under an optical microscope (Olympus CX31, Olympus Corporation, Tokyo, Japan)(Walkley and Black, 1934; Parr et al., 2001; Li et al., 2013a). The organic carbon in the phytolith was determined by an elemental analyzer (Model CHNS-O-Rapid, Heraeus, Germany). The PhytOC concentration (g kg^-1^) and PhytOC accumulation of the leaves (mg) was calculated using the following equations:


(1) $$\begin{array}{c} PhytOC\;concentration\;(g\;kg^{-1})=C\;concentration\;in\;phytoliths\;(g\;kg^{-1})\\ \times phytolith\;concentration\;(g\;kg^{-1})/1000 \end{array}$$

(2) $$PhytOC\;accumulation\;(mg)=PhytOC\;concentration\;(g\;kg^{-1})\times leaf\;biomass\;(g)$$

Soil total carbon (TC) and total nitrogen were determined by an elemental analyzer (Model CHNS-O-Rapid, Heraeus, Germany). Soil total silicon (TSi) was extracted by nitrate-lithium metaborate melting method and determined using the molybdenum blue colorimetric method (Lu, 2000) with an ultraviolet-visible spectrophotometer (UV-1800, Shimadzu, Japan). Soil pH was determined using a pH meter with soil to water ratio of 1:2.5 (m:m). Soil alkali-hydrolyzed nitrogen (AN) was determined by the alkaline diffusion method. Soil-available phosphorus (AP) was extracted with 0.05 mol L^-1^ HCl and 0.0125 mol L^-1^ H_2_SO_4_. and measured colorimetrically at 880 nm (PerkinElmer Lambda 45, PerkinElmer, USA). Soil available kalium (AK) was determined by ammonium acetate extraction with the flame photometry method, (Bao, 2000). Soil Total Kalium (TK) was determined by hydrofluoric acid digestion with the flame photometric method (NY/T 87-1988).

### 2.4 Statistical analysis

Statistical analyses were carried out with the SPSS 25.0 statistical software package (http://www-01.ibm.com/software/analytics/spss/). The mean values of all properties were calculated from the four replicates.

Effects of different treatments were compared by a one-way analysis of variance (ANOVA) and Fisher’s least significant difference (LSD) test at the P< 0.05 level of significance. Before the analysis of variance, the normality and homogeneity of variances across groups of the residuals were tested to make sure all the variables were satisfied the assumptions of ANOVA.

## 3 Results

### 3.1 Effects of different fertilization on accumulation of phytolith and PhytOC

**Figure 1:**
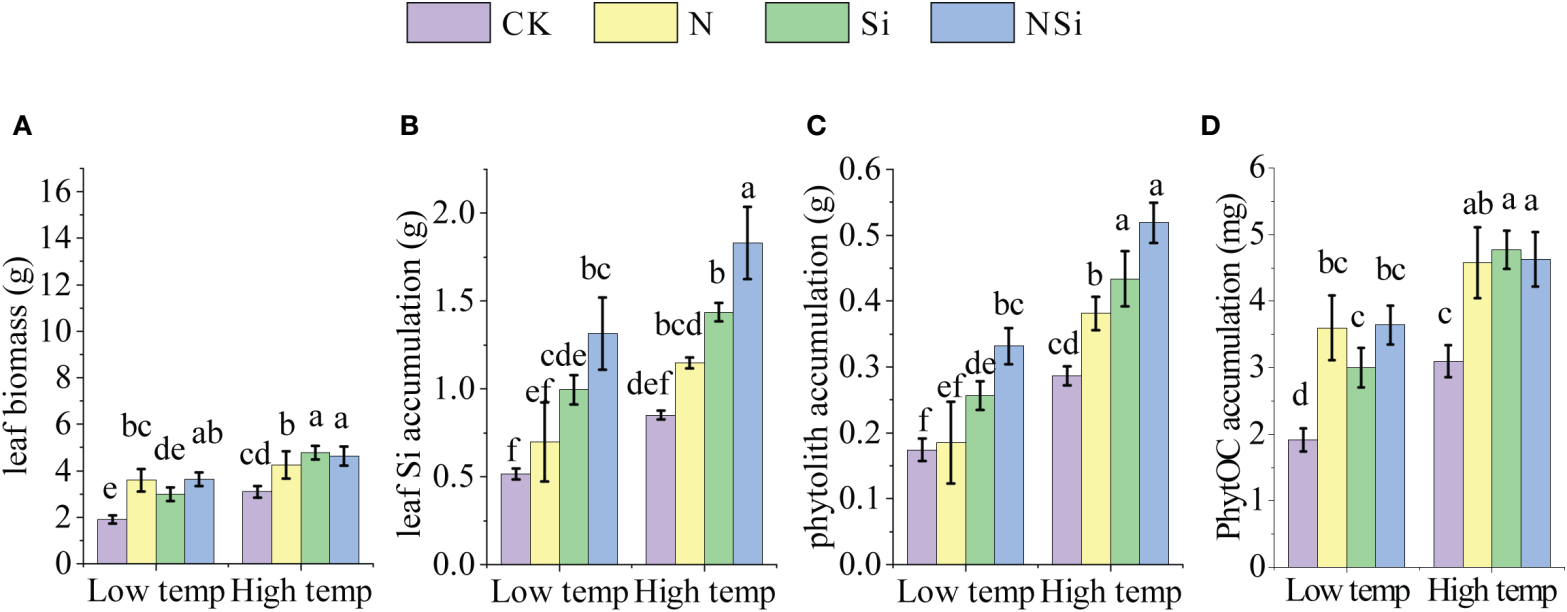
The leaf biomass, leaf Si accumulation, phytolith accumulation, and PhytOC accumulation of Moso bamboo leaves at low- and high temperature with different fertilization. **(A)** leaf Si biomass, **(B)** leaf Si accumulation, **(C)** phytolith accumulation, and **(D)** PhytOC accumulation. CK, N, Si, and NSi represent the level of fertilization at 0N 0Si kg/hm^2^, N157.5 kg/hm^2^, Si 315 kg/hm^2^, N157.5 kg/hm^2^ and Si 315 kg/hm^2^, respectively. Error bars represent the standard error of the means. Different letters indicate significant differences among the treatments of different fertilization at 0.05 level, respectively.

**Figure 1** shows the effects of fertilization on leaf biomass and the accumulation of leaf Si, phytolith, PhytOC under different temperatures. For the low-temperature group, under the N and NSi treatments, the leaf biomass of Moso bamboo increased significantly compared with CK. The increase in leaf biomass of Si treatment was insignificant for the low-temperature group. However, the application of Si fertilizer significantly increases the leaf biomass for the high-temperature group (**Figure 1A**). For both low- and high-temperature groups, the application of silicon fertilizer significantly increased the leaf Si accumulation compared with CK. In contrast, the difference in leaf Si accumulation between CK and the N treatment was insignificant (**Figure 1B**). For both low- and high-temperature groups, Si and NSi treatment significantly promoted the accumulation of phytolith and PhytOC compared with CK. Besides the N treatment, the accumulation of phytolith and PhytOC of the high-temperature group increased significantly compared with the low-temperature group (**Figures 1C, D**). In addition, the phytolith and PhytOC content for NSi is not necessarily increased compared with the Si treatment (**Figures 1C, D**).

### 3.2 PhytOC concentration at different growth period following different fertilization

**Figure 2:**
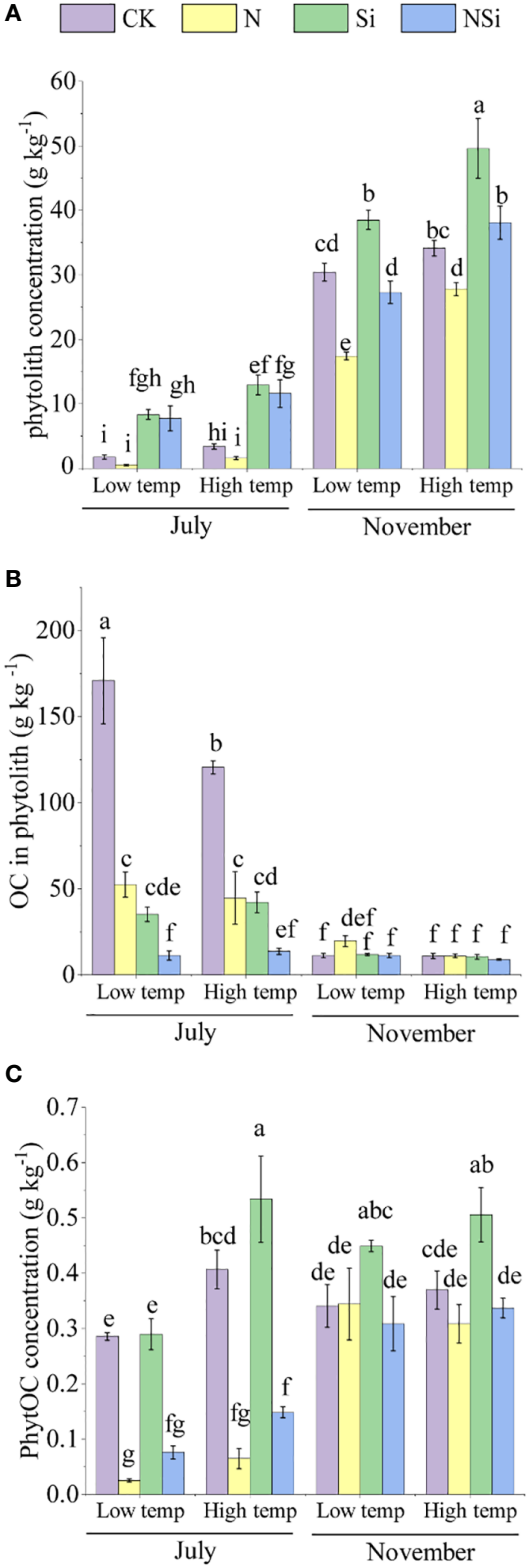
The concentration of **(A)** Moso bamboo leaves’ phytolith, **(B)** OC in phytolith, and **(C)** PhytOC with different fertilization at low and high temperature in July and November. CK, N, Si, and NSi represent the level of fertilization at 0N 0Si kg/hm^2^, N157.5 kg/hm^2^, Si 315 kg/hm^2^, N157.5 kg/hm^2^ and Si 315 kg/hm^2^, respectively. Error bars represent the standard error of the means. Different letters indicate significant differences among the treatments of different fertilization at 0.05 level, respectively.

The concentration of phytolith, OC in phytolith, and PhytOC across different growth periods is presented in **Figure 2**. In July, the concentration of phytolith in the Si and NSi treatments was significantly higher than in the CK and N treatments for both the low- and high-temperature groups. In November, however, the concentration of phytolith of Si treatment was significantly higher than the other treatments in each group (**Figure 2A**). For OC in phytolith, the CK treatment of both low- and high temperature groups had the largest concentration among the four treatments in July. In November, however, the difference in concentration of OC in phytolith was insignificant among the four treatments for both low- and high-temperature groups (**Figure 2B**). For concentration of PhytOC, the CK and Si treatment had a significantly larger concentration than N and NSi in both low- and high-temperature groups in July. In November, the Si treatment had the largest concentration of PhytOC among four treatments for both low- and high-temperature groups (**Figure 2C**).

### 3.3 Photosynthesis and transpiration induced by different fertilization

**Figure 3:**
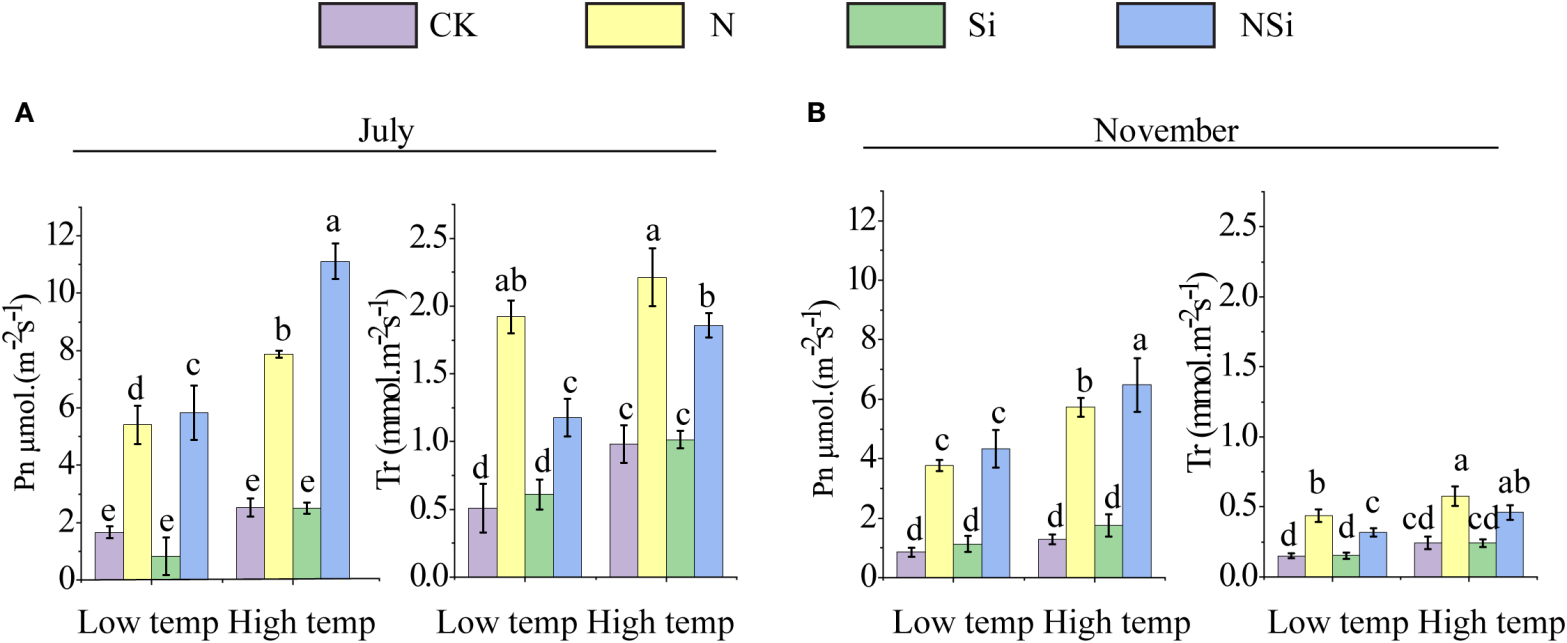
**(A)** photosynthesis and **(B)** transpiration of Moso bamboo leaves at low and high temperature in July and September. CK, N, Si, and NSi represent the level of fertilization at 0 N 0 Si kg/hm^2^, N157.5 kg/hm^2^, Si 315 kg/hm^2^, N157.5 kg/hm^2^ and Si 315 kg/hm^2^, respectively. Error bars represent the standard error of the means. Different letters indicate significant differences among the treatments of different fertilization at 0.05 level, respectively.

The photosynthesis and transpiration rates of low- and high-temperature groups in July and November are given in **Figure 3**. For both low- and high-temperature groups, the application of nitrogen fertilizers (including N and NSi) significantly promoted photosynthesis and transpiration rates in July. The difference in photosynthesis and transpiration rate between the CK and the Si treatments was insignificant (**Figure 3A**). This is also true for low- and high-temperature groups in November (**Figure 3B**).

### 3.4 Accumulation of phytolith and PhytOC following Moso bamboo growth

**Figure 4:**
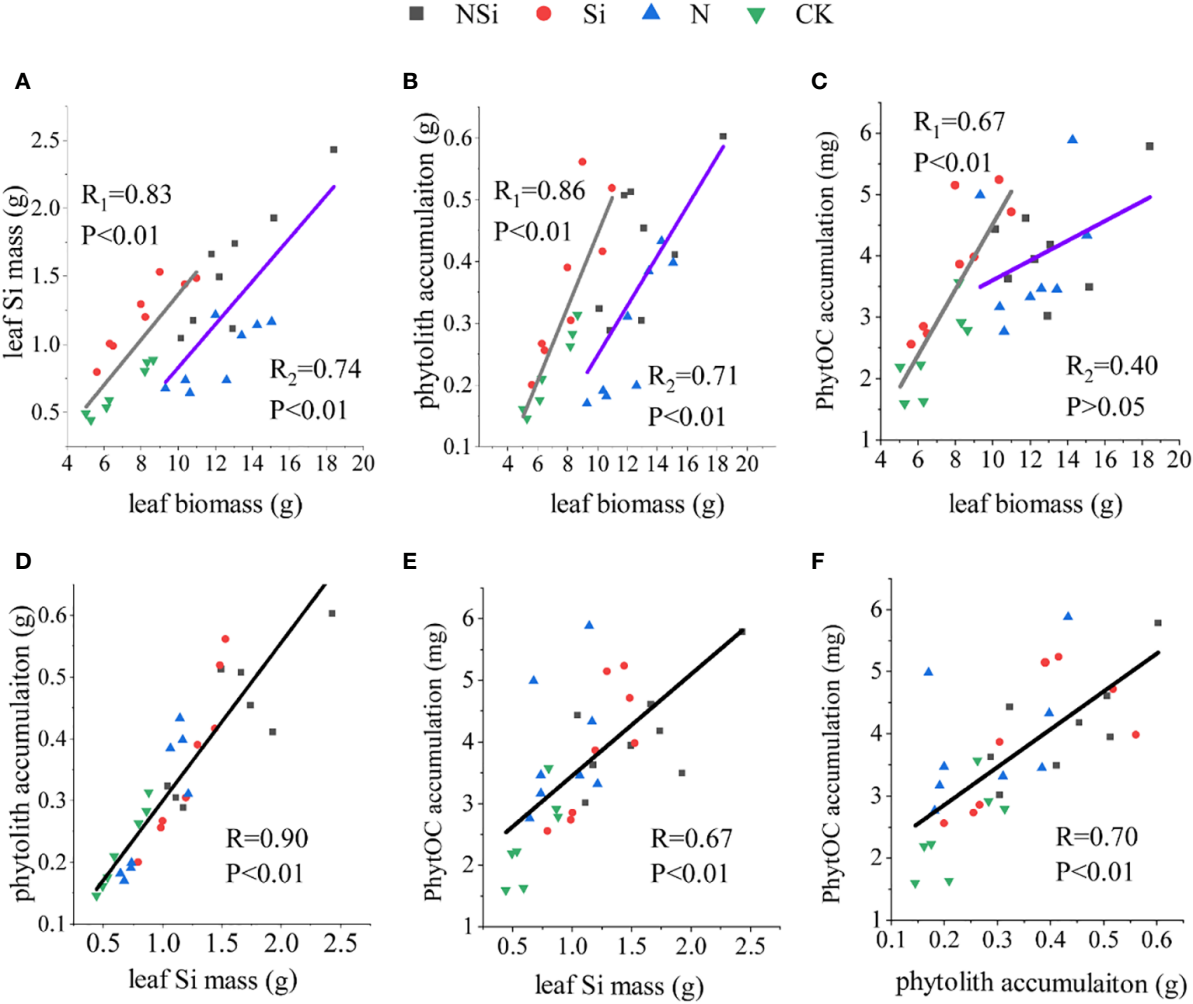
Correlation of **(A)** biomass with Si mass, **(B)** biomass with phytolith accumulation, **(C)** biomass with PhytOC accumulation, **(D)** Si mass with phytolith accumulation, **(E)** Si mass with PhytOC accumulation, and **(F)** phytolith accumulation with PhytOC accumulation in Moso bamboo leaves. CK, N, Si, and NSi represent the level of fertilization at 0N 0Si kg/hm^2^, N157.5 kg/hm^2^, Si 315 kg/hm^2^, N157.5 kg/hm^2^ and Si 315 kg/hm^2^, respectively. R_1_ represents the correlation coefficient between Si fertilizer and NSi fertilizer treatment. R_2_ represents the correlation coefficient between N fertilizer and CK treatment.

The relations between leaf Si mass and leaf biomass are shown in **Figure 4A**. Liner fitting revealed that CK and Si treatments had the same slope as the N and NSi treatments. However, the interception of CK and Si treatment was larger than that of N and NSi. For the relations between phytolith accumulation and leaf biomass, the slope of CK and Si treatments was larger than that of N and NSi treatment, whereas the reverse was true for the interception (**Figure 4B**). The overall pattern of leaf biomass versus PhytOC accumulation (**Figure 4C**) was similar, as shown in **Figure 4B**; however, a comparison of slope and interceptions was not possible because the liner fitting of N and NSi was not significant (**Figure 4C**). The accumulation of phytolith and PhytOC increase with the increase of leaf Si mass (**Figures 4D, E**). Our findings further revealed that PhytOC accumulation increased as phytolith accumulation increased (**Figure 4F**).

## 4 Discussion

### 4.1 Factors related to the PhytOC accumulation in Moso bamboo leaves

Besides fertilization, the accumulation of PhytOC in Moso bamboo leaves could be affected by many factors, including the quality of the Moso bamboo seedlings, the type of soil, and cultivation conditions (mainly temperature in our study). Based on our results, we infer that the PhytOC accumulation quality was not attributed to the Moso bamboo seedlings, because the differences in properties of Moso bamboo seedlings for different temperature groups and fertilization treatments are insignificant (**Table 2**). The contribution of soil type to the PhytOC accumulation was also excluded because of the cultivation of all the Moso bamboo seedlings using the same soil. Temperature is one of the critical factors that affect the accumulation of PhytOC (**Figures 1**, **4A–C**). Higher temperatures significantly increase the accumulation of PhytOC. This could attribute to the increase in metabolite production in the bamboo leaves under higher temperatures. These metabolite productions were captured and wrapped by phytolith as PhytOC. Thus, the accumulation of PhytOC in the high-temperature group was significantly larger than that of the low-temperature group. Based on the discussion above, it is inferred that the increase of the temperature is another effective method for promoting PhytOC accumulation because compared with low-temperature group, the accumulation of PhytOC for the high-temperature group increased by 61.1% even without fertilization. Therefore, the bamboo forests in warmer areas are more potential for long-term carbon sequestration. Moreover, our results further indicate that global warming also poses a positive effect on the PhytOC accumulation, which automatically mitigates climate change by sequestering more carbon in the phytolith.

### 4.2 Possible mechanism of PhytOC accumulation following different fertilization

The effects of nitrogen application were revealed by the interception of the fitting curves with (N and NSi) and without nitrogen treatment (CK and Si), respectively. The shift of the interception indicates the marked increase of leaf biomass following nitrogen application. Moreover, the application of silicon fertilizer (both Si and NSi) increased not only the leaf Si accumulation but also the accumulation of phytolith and PhytOC (**Figures 4B, C**). The beneficial effects of fertilization on PhytOC accumulation were also reported in previous research (Li et al., 2013b; Guo et al., 2015; Zhao et al., 2016; Huang et al., 2020). The slope of the fitting curves generally revealed the biological dilution effect on the concentration of leaf Si, phytolith, and PhytOC (**Figures 4A–C**). In spite of the marked increase of leaf biomass, the identical slope in **Figure 4A** indicating the dilution effect on leaf Si concentration following different fertilization is unobservable. In contrast, the dilution effect was clearly observed in **Figure 4B**, indicating the increase of leaf biomass following nitrogen application (N and NSi) diluting the concentration of phytolith in the Moso bamboo leaves during the growth process.

Our results further indicated bio silicification process of Moso bamboo is formed by a completely passive mechanism because the accumulation of phytolith and leaf Si is affected directly by the concentration of soil-available silicon. This was also supported by previous research (Exley, 2015). The phytolith is mainly produced within the bamboo leaves, which relies on the amount of silicon absorption (Parr and Sullivan, 2005; Parr et al., 2010; Song et al., 2016). Thus, the application of silicon fertilizer is beneficial to phytolith accumulation, which was supported by numerous studies (Song et al., 2012; Ying et al., 2016; Ru et al., 2018; Yang et al., 2018; Lu et al., 2021). Fertilization also affects the photosynthesis and transpiration rate of Moso bamboo leaves, especially for the N and NSi treatment, because About 75% of leaf N is allocated in the chloroplasts, and most of them are used photosynthetic apparatus (Gao et al., 2016). The increase in photosynthesis and transpiration rate for the N and NSi treatment could also attribute to the increase in the total leaf area that is related to the leaf biomass after the nitrogen fertilizer was applied (**Figures 1D**, **3**).

### 4.3 Fertilization strategies for promoting the PhytOC accumulation

The PhytOC is critical for long-term carbon sequestration. Therefore, increasing the accumulation of PhytOC is the priority principle for establishing management strategies. According to our study, two primary factors can enhance the accumulation of PhytOC, including fertilization and temperature level. For fertilization, the differences in the accumulation of PhytOC among all three fertilizers are insignificant. Therefore, the economic benefit-cost ratio should be considered when selecting fertilizers. As one of the most important economic forests, nitrogen fertilizer was widely applied for increasing the biomass of the Moso bamboo forest. Therefore, the application of nitrogen fertilizer is a simple and effective method that is suitable for enhancing PhytOC accumulation. Silicon fertilizer is usually optional in the management of the Moso bamboo forest. Although the silicon fertilizer can significantly increase the accumulation of PhytOC. However, the application of silicon fertilizer is limited by multiple factors. One of the limiting factors is the content of soil available silicon, which varies differently in the different Moso bamboo growing regions (Hu et al., 1994). Moreover, the effectiveness of the silicon fertilizer relies on soil pH. Thus, the application of silicon fertilizer is not a practical method for promoting PhytOC sequestration. The combination of Si and N didn’t gain extra benefits on PhytOC accumulation. Therefore, the synergistic use of Si and N fertilizer is not an optimized management method for enhancing the PhytOC accumulation.

## 5 Conclusions

This study investigates the effects of temperature elevation and different fertilization on the accumulation of phytoliths and PhytOC. Our results indicated that the accumulation of PhytOC in Moso bamboo leaves positively responds to temperature elevation and fertilization. In warmer areas, the higher temperature significantly increases the accumulation of PhytOC. For routine forest management strategies, the application of nitrogen fertilizer is a practical and effective method for promoting long-term carbon sequestration for the Moso bamboo forest. Based on our study, it is predicted that global warming poses a positive effect on the long-term carbon sequestration of Moso bamboo, which enables the automatic mitigation of climate change. Given the promoting strategies of PhytOC for mitigating climate change, the application rate of fertilizers and the interaction between fertilizers and temperatures will be taken into consideration when establishing fertilization strategies in our future studies.

## Data availability statement

The original contributions presented in the study are included in the article/supplementary material, further inquiries can be directed to the corresponding author/s.

## Author contributions

LL and XY were involved in the manuscript writing, CH and PJ participated in the experimental design, SC and YZ were involved in providing advice on the writing of the manuscript. LL and YJ completed the experiment. All authors contributed to the article and approved the submitted version.
